# Mad Cows and Cooked Geese

**DOI:** 10.1100/tsw.2001.4

**Published:** 2001-01-26

## Abstract

Science leads with the results of a recent gloomy assessment of the cause of global climate change. Nature leads off this week with a story about U.S. attempts to prevent the spread of mad cow disease.

It is not the sun, natural climate fluctuations or a bug in a computer program, reports *Science* in their lead story, it's us. Human beings are cooking their own goose in a globally warming stew of greenhouse gas emissions. *Nature* also covered this story in their number nine spot. According to the *Science* story, the United Nations-sponsored Intergovernmental Panel on Climate Change (IPCC) officially declared early this week that "most of the observed warming over the last 50 years is likely to have been due to the increase in greenhouse gas concentrations." The previous IPCC report in 1995 (the first five-year report was released in 1990, reports *Nature*) cautiously accused human-produced greenhouse emissions of causing the observed rise in global temperatures, but several scientific loopholes forced them to equivocate. Five years later, both journals report, those loopholes have been cinched shut like a noose around our neck. The report concludes that the average global temperature is rising, humans are causing the increase, and the global fever could spike up by anywhere from a modest 1.4°C to "staggering" 5.8°C by the year 2100. Socioeconomic uncertainties account for most of the range of estimates, i.e. whether or not governments, industries, and individuals will change their behavior. And one individual with close government and industry connections is under particularly close scrutiny: U.S. President George W. Bush.

In other international news, U.S. government agencies are working hard to keep the rising European tide of bovine spongiform encephalopathy (BSE), more commonly known as mad cow disease, from washing up on the shores of the United States, reports *Nature* in their lead story. Mad cow disease is extremely rare in Europe. *Nature* reports that only 80 people have died from variant Creutzfeld-Jakob disease (vCJD), an analogous human disease presumed to be contracted by eating infected cattle. And it has never been detected in the U.S., reports *Nature*. But now that Italy has joined the United Kingdom, Switzerland and Ireland on the dubious list of countries with confirmed cases of BSE, Americans are realizing that they are not immune from the spreading disease, says *Nature*. Rising public awareness has in turn placed enormous pressure on U.S. regulatory agencies to keep BSE out of the country. That is easier said than done.

Although the U.S. has imported no live U.K. beef since 1985, reports *Nature*, tonnes of "high-risk European beef" are still flowing across the Atlantic. It just doesn't look like a cow. Gelatin, collagen, semen, and albumin from beef are widely used in imported products like cosmetics and vaccines, reports *Nature*. Illustrating both the strong desire to act and the deep uncertainty about what to do, the U.S. last week banned blood donations from anyone who has, since 1980, spent ten years in France, Portugal or the Republic of Ireland, says the *Nature* article, even though no one knows if either BSE or vCJD can be transmitted through transfusions. *Science* did not cover this story.
